# Birth Timing for Mountain Lions (*Puma concolor*); Testing the Prey Availability Hypothesis

**DOI:** 10.1371/journal.pone.0044625

**Published:** 2012-09-24

**Authors:** Brian D. Jansen, Jonathan A. Jenks

**Affiliations:** Department of Natural Resource Management, South Dakota State University, Brookings, South Dakota, United States of America; Université de Sherbrooke, Canada

## Abstract

We investigated potential advantages in birth timing for mountain lion (*Puma concolor*) cubs. We examined cub body mass, survival, and age of natal dispersal in relation to specific timing of birth. We also investigated the role of maternal age relative to timing of births. We captured mountain lion cubs while in the natal den to determine birth date, which allowed for precise estimates of the population birth pulse and age of natal dispersal. A birth pulse occurred during June–August. Body mass of cubs was related to litter size and timing of birth; heaviest cubs occurred in litters of 2, and those born after 1 July. Cubs born within pulse months exhibited similar survival to those born out of the pulse. We found that cubs born April–June dispersed at younger ages than those born after 1 July. There was less variation in birth timing for 1^st^ litters of females than older females. We hypothesize that cubs born after the peak in births of neonate prey are advantaged by the abundance of vulnerable prey and those cubs and mothers realize an evolutionary advantage.

## Introduction

Pattern of births for mountain lions (*Puma concolor*) is better characterized as a pulse over several months [1] than a sudden peak, characteristic of many ungulate populations [2]. Studies of mountain lions over numerous years have documented litters born in every month of the year [3]; however, the majority of births occur from June through October in North America [4]. Logan and Sweanor [1] hypothesized that because the pattern coincided with availability of young ungulates, that cubs born within the pulse had a greater chance of survival. However, Laundré and Hernández [4] tested this prediction and concluded that there were no differences in survival related to timing of birth.

Mountain lion cubs weigh about 400–500 g at birth [3]. Males typically outweigh females throughout their lives [1]. Litter sizes range from 2 to 4 cubs [1]. Despite existing information on reproductive ecology of mountain lions, the extent to which number of cubs in a litter influences birth weights is unknown.

No study has sought to elucidate the age of dispersal in relation to the timing of birth, although geographic patterns of juvenile dispersal in mountain lions are adequately documented [5]–[8]. Juvenile mountain lions disperse about 1 month after becoming independent of mothers [7], [8], and both sexes become independent at similar ages [1]. Males disperse farther than females [1], [8], [9].

The birth pulse in mountain lions has been previously investigated by summing all litters born regardless of maternal age or experience [1], [4]. Parturitions have been documented an average of 3 months (mean birth interval - mean age of independence [17 - 13.7 = 3.3 months]) following departure of successful litters [1]. Mean gestation length for mountain lions is 3 months [1], thus subsequent litters begin close to departure of previous successful litters. Females that lost litters before cubs reached independence produced litters from 4–10 months later [1], suggesting a breeding lapse of 1–7 months following unsuccessful litters. Introduction of these variables into the birthing schedule suggests that birth timing through the year will become less predicable as females age.

Our objective was to determine if differences occur in body mass at 1 week of age, survival to 1 year, and age of natal dispersal for mountain lions with respect to timing of birth. If timing of births is related to prey availability [1], then we expect a birth pulse (highest frequency of litters born) coincident with availability of neonate ungulates. We expect heavier cubs and higher survival for those cubs born within the pulse. However, natal dispersal is thought to be related to timing of the mother's estrous cycle [1]; thus, we predict no relation of timing of birth to age of dispersal. We predict that birth timing in younger females or first litters will be more precise than birth timing in older females of subsequent litters, because timing of subsequent litters depends on completion (i.e., mortality, independence) of the previous litter.

## Results

### Birth pulse

**Figure 1:**
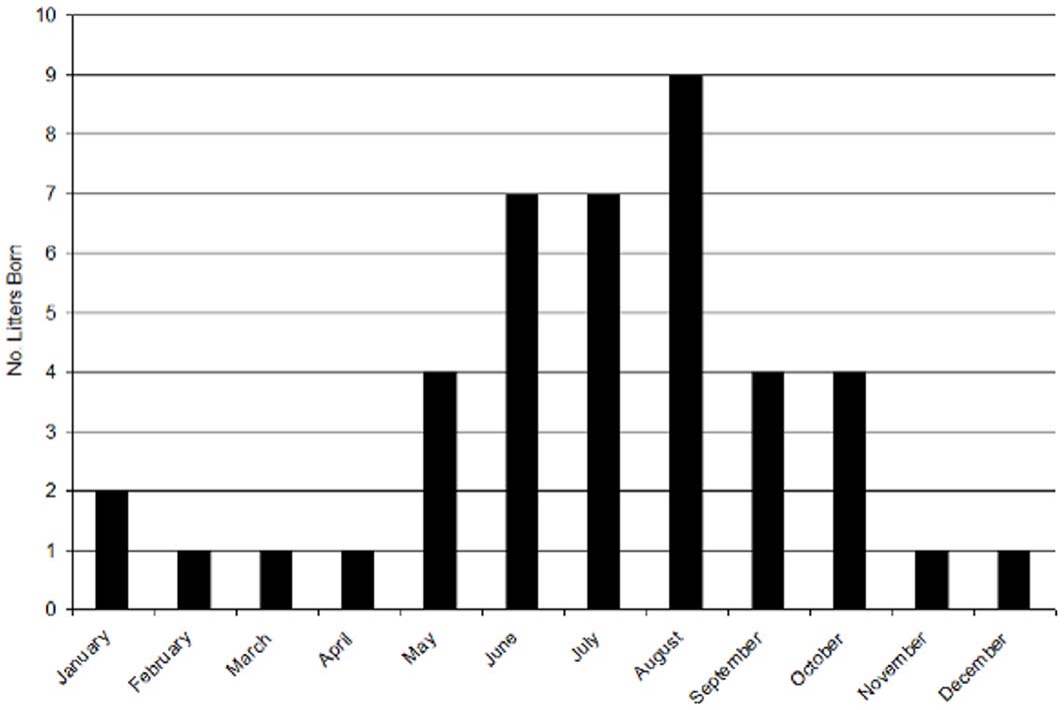
Number of litters born during each month from 2005 to 2009 for mountain lions (*Puma concolor*) in the Black Hills, South Dakota, USA.

We captured 42 litters from 2005 to 2009. We documented litters born in every month; however, frequency of litters produced was not uniformly distributed through the year (Fig. 1, *Χ* ^2^_11_ = 25.43, *P*<0.01). Months with higher than expected births included the period June–August; thus, we defined our birth pulse as occurring from 1 June to 31 August. We further delineated the year as spring (April–May), early pulse (June), middle pulse (July), late pulse (August), autumn (September–October) and winter (November–March, Table 1).

**Table 1:** Environmental characteristics exhibited during annual periods and the number of documented mountain lion (*Puma concolor*) cubs born during each period in the Black Hills, South Dakota, USA.

Period	Months	No. cubs	Environmental Characteristics
Spring	April–May	12	Variable snowfall and cold temperatures; adult-sized ungulates with relative lowest availability
Early	June	13	Warm days, cool nights; neonate ungulates present, but in “hider" phase resulting in low availability
Middle	July	15	Warm days, warm nights; neonate ungulates present and in “flee" phase resulting in highest availability
Late	August	16	Warm days, warm nights; adult and juvenile ungulates present resulting in high availability
Autumn	September–October	13	Cool days; cool nights; adult and larger juvenile ungulates present resulting in high availability
Winter	November–March	4	Snow; cold days, cold nights; adult-sized ungulates available, post-hunting season results in declining availability and harsh environmental conditions.

### Cub body mass

We used data for 74 (34M, 40F) cubs originally captured in natal dens, at ages ranging from 1–4 weeks, to estimate influence of birth timing on weight at 1 week of age. We found few cubs born during winter (*n* = 4), so we censored those animals from analyses regarding cub weights. We found that timing of birth (*F*_1,61_ = 3.850, 2-sided *P* = 0.016) and litter size (*F*_1,61_ = 3.801, 2-sided *P* = 0.056) influenced body mass of cubs at 1 week of age, whereas sex (*F*_1,61_ = 0.624, 2-sided *P* = 0.866) had no effect. Mean body mass for male (*n* = 34) and female (*n* = 40) cubs at 1 week of age was 1.162 kg±0.112 (SE) and 1.102 kg±0.095, respectively.

There was a declining trend in body mass as litter size increased. Mean body mass of cubs in 2-, 3-, and 4-cub litters were 1.114 kg±0.135, 1.053 kg±0.091, and 0.912 kg±0.089, respectively. Cubs born in litters of 2 were 22% larger at 1 week of age than cubs in litters of 4 (Extra sums-of-squares *F*_1, 68_ = 3.308, 2-sided *P* = 0.146).

After accounting for sex and litter size, mean mass of cubs born within the birth pulse (June–August) was similar to cubs born outside the birth pulse (Extra sums-of-squares *F*_1, 61_ = 2.40, 2-sided *P* = 1.0); however, a trend existed, with cubs born in spring lightest and body mass of cubs increasing through autumn (Extra-sums-of-squares *F*_1, 62_ = 3.98, 2-sided *P* = 0.102). Cubs born during spring and early pulse averaged 230 g±72 (SE) lighter in mass than cubs born during the middle pulse through autumn (Extra sums-of-squares *F*_1, 60_ = 12.302, 2-sided *P* = 0.002).

When we reevaluated birth timing relative to ungulate birth periods, we found a strong relationship to timing of birth and weight at 1-week of age (*F*_1,61_ = 4.310, 2-sided *P* = 0.004). Cubs born before the ungulate birth peak (*n* = 10) weighed 869 g (mean)±96 (SE) whereas cubs born coincident with ungulate birth peaks and 30 days following (*n* = 20) weighed 934±109 g. Those cubs born 30–60 days after the ungulate birth peak (*n* = 31) weighed 1,174±100 g, whereas cubs born >60 days after the ungulate birth peak (*n* = 13) weighed 1172±102 g.

### Cub survival

**Figure 2:**
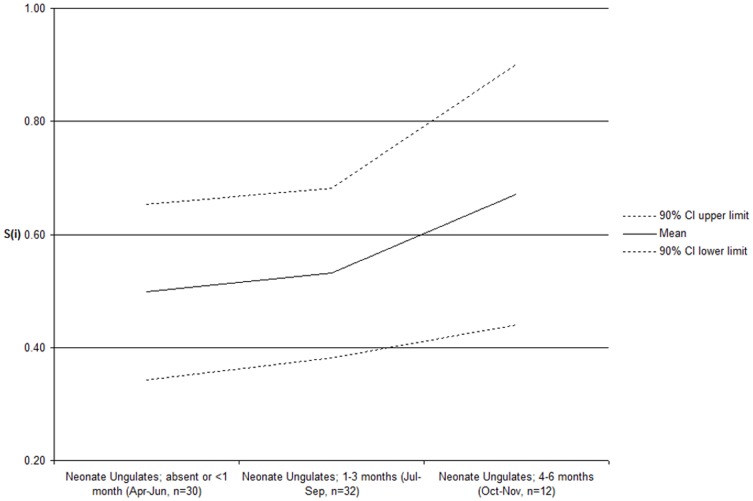
Mountain lion (*Puma concolor*) cub survival in relation to neonate ungulate presence and age in the Black Hills, South Dakota, USA.

No difference was documented in sex-specific survival (Male, *n* = 42, *S*_(i)_ = 0.55, SE = 0.08; Female, *n* = 36, *S*_(i)_ = 0.58, SE = 0.08; 2-sample *t*_76_ = 0.264, 2-sided *P*>0.50); therefore, we pooled sexes in subsequent analyses. We found no support (evidenced by overlap in confidence intervals) for our hypothesis that survival was greater for cubs born within the birth pulse (*n* = 47, *S*_(i)_ = 0.55, 95% C.I. = 0.41–0.69) compared to those born outside the pulse (*n* = 31, *S*_(i)_ = 0.58, 95% C.I. = 0.41–0.74). However, we found an increasing trend in survival relative to prey abundance and vulnerability (Fig. 2). Cubs born before the ungulate birth period or <30 days afterward (April–June, *n* = 30, *S*_(i)_ = 0.50, SE = 0.09) had mean survival rates lower than those born 30–60 days after the ungulate birth peak, but before winter (Jul–Sep, *n* = 32, *S*_(i)_ = 0.53, SE = 0.09; Oct–Nov, *n* = 12, *S*_(i)_ = 0.67, SE = 0.14).

### Dispersal age

We obtained dispersal ages for 22 juvenile mountain lions (14M, 8F). Male mountain lions dispersed at a mean age of 14.7 months±0.84 (SE) and females dispersed at 15.3±1.15 months. While accounting for variation in timing of birth, we found no sex-related differences in natal dispersal age (*F*_1,20_ = 1.420, 2-sided *P* = 0.50). There was evidence that cubs born during the birth pulse dispersed when 2.5 months younger (±1.3 SE) than those born outside the pulse (Extra sums-of-squares *F*_1, 17_ = 3.92, 2-sided *P* = 0.120). However, there was stronger evidence (Extra sums-of-squares *F*_1, 15_ = 8.971, 2-sided *P* = 0.018) that cubs born during spring, early-, and mid-pulse (1 April–30 July, *n* = 9) dispersed 4±1.3 months younger than cubs born in the last month of the birth pulse and winter (1 August–31 January, *n* = 13). When we excluded cubs born in July (i.e., middle month of pulse), there was strong evidence of a mean 4±1.5 month difference in dispersal ages (Extra sums-of-squares *F*_1, 15_ = 7.405, 2-sided *P* = 0.032).

All cubs that were born in early or mid-pulse dispersed during the summer (June–August) or autumn (September–November) after attaining an age of 1-year. All cubs born late in the birth pulse dispersed during winter (December–February) or spring (March–May) after becoming 1-year of age.

### Mother and first litters

**Figure 3:**
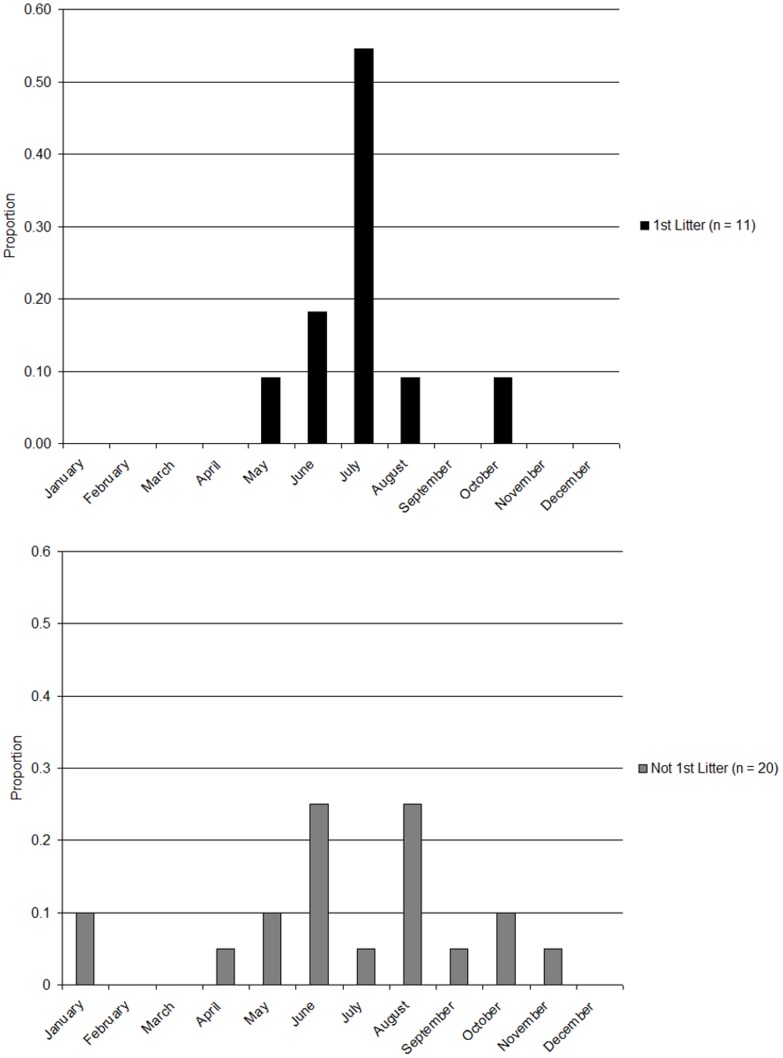
Frequency of births by month for first and non-first litters of adult female mountain lions (*Puma concolor*) in the Black Hills, South Dakota, USA.

We documented 31 litters that were born to females that we had recorded evidence of previous nursing [25]. Birth timing period was narrower for females that had not previously nursed cubs (*n* = 11) and occurred mostly in July (*n* = 6), and few occurred in June or August (*n* = 3, Fig. 3). Birth timing for females that had previous litters (*n* = 20) were dispersed throughout the year, while still exhibiting a pulse in June–August (Fig. 3).

## Discussion

Logan and Sweanor [1] hypothesized that cub survival might be higher for cubs born within the birth pulse, than those born outside the pulse, because the pulse in their central New Mexico study area generally coincided with ungulate birthing periods. Laundré and Hernández [4] summarized available data on birth frequencies and concluded that indeed the pattern across North America suggested a birth pulse that occurred anywhere between June and October. They further tested if cubs born in their Idaho study area within the birth pulse documented by Logan and Sweanor ([1]; July–September) exhibited higher survival; no difference was documented. We also did not document differences in survival between pulse-born and other cubs, as well. These results might not be surprising, given the ability of mountain lions to kill prey animals throughout the year. Providing sufficient quantities of food for cub survival (e.g., maintenance) would require less prey than to provide for individual quality (e.g., body mass, growth rates). Clearly, energy requirements for maintenance are less than for growth and reproduction [10]. Although survival tended to increase after neonate ungulates switched to the “fleeing" strategy, the use of cub survival might not be a sensitive metric to evaluate reasons for timing of birth in mountain lion populations. Because cubs are dependent on an adept predatory mother to provide food for survival until independence, and mortality is often caused by other mountain lions [1], survival might be a poor indicator for variation in reproductive quality of birth timing.

We found evidence that there were other advantages in timing of birth. Timing of birth predominantly influenced body mass of cubs and the dispersal age of juveniles. However, the benefits seemed to be best tested by using actual ungulate availability, as predicted by Logan and Sweanor [1], than basic frequency of births (i.e., birth pulse) as tested by Laundré and Hernández [4]. We found that cubs born 1 April to 30 June were smaller and dispersed at an earlier age than cubs born 1 July to 31 January in our study area, even though June coincided with ungulate births.

More cubs were born during July and August (40% of all litters) than any other 2-month period, which occurred >30 days after the births of ungulate neonates in our study area. Neonates of white-tailed deer (*Odocoileus virginianus*), mule deer (*O. hemionus*), and elk (*Cervus elaphus*) exhibit a scent-less hiding strategy for 2–4 weeks, presumably to avoid predators until they are mobile enough to flee [11]–[14]. Because of this hiding strategy, neonates might be less vulnerable to predation, by sight predators such as mountain lions, during the hiding-phase; although, the strategy might be less effective for animals with highly developed olfactory senses (i.e., Canidae [15]; Ursidae [16]). The abundance of vulnerable prey [17] during July and August, should allow mothers to more easily find food with less travel and spend more time at dens providing food for their young cubs. In fact, we found cubs born >30 days after the ungulate birth peak were 21% larger, at 1 week of age, than cubs born before this period. Cubs born prior to or during the ungulate birth peaks necessarily exhibit lower developmental growth, because prey availability is lowest at this time of the year.

We suggest that there are advantages to birth timing for mountain lions. Birth following the birth peak for ungulates in North America leads to heavier body mass and older dispersal. Subadults dispersing at 17 months (born after the switch) are presumably heavier and more experienced, than those dispersing at 13 months (born before the switch). Some of the advantages might influence survival through the subadult life-stage, which translates to an evolutionary advantage for both mother and offspring. Thus, births of mountain lions should trend toward a narrower as opposed to wider birthing period.

Because of the stochastic nature of most cub mortalities [1], [18], timing of first litter should show less variation if it is inherent or in relation to resource availability because timing of subsequent litters are related to completion of a previous litter. We showed that timing of birth is more narrowly concentrated, in time, for 1^st^ time mothers than for older mothers. These data are in accordance with the prediction that birth timing is related to prey availability [1].

However, breadth of the population birth pulse is maintained by variation in reproductive maturity, birth intervals, and the vagaries of mortality. Logan and Sweanor [1] found mean age of first litter of 29 months and mean birth intervals of 17 months, thereafter. Natural mortality of cubs is dominated by infanticide by other mountain lions, which likely occur during chance encounters. Mothers that lose cubs can rebreed as soon as 23 days following mortality event and give birth 113 days following the mortality event [1]. Despite the predictability of ungulate birth peaks and subsequent timing of anti-predator strategy switch, none of the reproductive traits for mountain lions promote the persistence of a strict annual birth peak. No study has evaluated the possibility of variation in pulse dates within populations that depend upon the age structure of the female population.

We offer a hypothesis and predictions regarding the birth pulse in mountain lions and recommend testing with data from other populations. We hypothesize that mountain lion births are adaptively timed closely to the availability of primary prey in environments, where primary prey exhibit predictable birth peaks, but that reproductive biology and random mortality events after the initial litter result in a broadened birth pulse that adheres less strongly to the timing of prey availability. We predict that in environments where the primary prey for mountain lions exhibit “hider" predator-avoidance strategies (e.g., deer, elk) and where timing of ungulate birth peaks is predictable (e.g., north/south temperate latitudes) that first litters of mountain lions will be closely timed to follow the switch from “hider" to “fleer" by neonates. We also predict that in environments where primary prey species do not exhibit a “hiding" anti-predator strategy as neonates are capable of fleeing shortly after birth (e.g., bighorn sheep [*Ovis canadensis*], guanaco [*Llama guanacoe*]), yet birth peaks are predictable (e.g., non-desert systems in North America, temperate South America), that first litters of mountain lions will be closely timed to the birth peak of those primary prey species. We predict that in environments where prey are not born in predictable peaks or the sum of births in multiple primary prey species provide abundant neonates throughout the year (e.g., peccary [*Pecari* spp.], Sonoran Desert, tropical regions), that mountain lions will not exhibit a birth pulse or the pulse will be broader than 3–4 months, characteristic of populations studied in North America [4].

## Materials and Methods

### Ethics Statement

Our capture and handling procedures followed guidelines of the American Society of Mammalogists [19] and were approved by the Institutional Animal Care and Use Committee (Approval No. 07-A024) at South Dakota State University.

### Study area

We studied mountain lions in the Black Hills ecoregion (N44.09375°, W103.77691°) of South Dakota, USA between 2006 and 2009. The Black Hills are a complex of forested ridges, valleys, and steep canyons [20]. Climate patterns in the Black Hills are characterized by hot summers and cold winters typical of a continental climate regime. The plant community is dominated by ponderosa pine (*Pinus ponderosa*) forests, but also contains spruce (*Picea glauca*), aspen (*Populus tremuloides*), birch (*Betula* spp.), and oak (*Quercus macrocarpa*) trees [21]. Potential ungulate prey included white-tailed deer (*Odocoileus virginianus*), mule deer (*O. hemionus*), elk, American bison (*Bison bison*), mountain goat (*Oreamnos americanus*), and bighorn sheep as well as domestic livestock species. No other large carnivores were present in this system. Bobcat (*Lynx rufus*) and coyote (*Canis latrans*) are mesocarnivores that occur sympatrically with mountain lions.

*Field Methods*.—We used foot-snares [22], trained hounds [23], and cage traps [24] to capture adult female (>3 yrs) mountain lions throughout the year. We used a mixture of telazol and xylazine and administered them at recommended dosages [25]. We aged mountain lions by tooth wear and pelage characteristics and noted previous reproductive activity [26]. We outfitted each mountain lion with a radiocollar (MOD-500, Telonics, Mesa, Arizona, USA) and relocated radiocollared mountain lions 1-time per week by using homing-telemetry techniques from a fixed-wing aircraft [27]. We used homing techniques while on foot for females where ≥2 aerial telemetry locations were within 500 m of each other over a 3-week period. Closer investigations normally revealed adult female mountain lions located within nursery dens [1], [28] with newly-born cubs. We captured all cubs by hand without chemical immobilization, often while the mother was away from the site [1]. We determined sex and age of cubs by tooth eruption and appearance of eyes (e.g., open or closed; [29]). Based on tooth eruption patterns and relatively young age of cubs, birth dates were within a few days to <2 weeks of the true birth date. At the time of capture, we gave each cub a unique tattoo and some were radiocollared with small, expandable collars (MOD-125, Telonics).

When cubs were approximately 9 months old, we recaptured them by using the same techniques as those used for adult mountain lions and refitted cubs with larger radiocollars (MOD-500, Telonics) or global positioning system (GPS) collars (single D-cell, Northstar, Virginia, USA). We used fixed-wing aircraft or satellite-telemetry to relocate subadults and mothers. When a juvenile mountain lion left the natal area, we recorded the mid-date between the last location in the natal area and the first location outside the natal area. In some cases, dispersing mountain lions made exploratory movements and returned to the natal area and subsequently left again or established a home area at least partly overlapping the natal area (e.g., female philopatry). We recorded dispersal age as the mid-date the juvenile made the initial exploratory movement, regardless of subsequent return to the natal area. If an animal was not located outside the natal area until the animal became 3 years old, we censored the animal from analyses. These animals were censored because exploratory movements can be of short duration [30], [31] and frequency of weekly telemetry flights might not have detected exploratory movements.

We plotted all birth dates and defined the birth pulse as the period where ≥40% of births occurred and number of births were higher than expected [1], [4]. We delineated the births as within or outside birth pulse months and also as early (1^st^ one-third), middle (central one-third), late (final one-third), and outside pulse. In addition, we delineated cub births as before ungulate birth peak (before 1 June), before neonate ungulates switch predator avoidance strategies (hiding vs. fleeing [11], [14]; 1 June–23 June), <60 days after strategy switch (23 June–23 August), >60 days after strategy switch (23 August–23 October), and winter (December–February). In our study area, deer and elk dominate the diets of mountain lions (Smith and Jenks, South Dakota State University, *unpublished data*). The birth peak for mule deer was 7–14 June [32], for white-tailed deer it was 7–17 June [33], and for elk it was 28 May–4 June (Schmitz 2010, South Dakota Game, Fish, and Parks, *unpublished data*).

### Analysis

We used JMP 8.0 (SAS Institute, Cary, North Carolina, USA) statistical software to perform statistical analyses. We used chi-squared test for independence to determine if the monthly distribution of litters was uniform through the year [1], [4]. We used MANOVA to examine the influence of birth timing on body mass and age of dispersal of mountain lions, while accounting for other potential influences (i.e., sex, litter size, age at capture). We used extra sums-of-squares *F*-tests within a MANOVA framework to test for differences in birth timing, while accounting for differences in sex, litter size, and age at capture. We used a known-fates model in Program MARK [34]–[36] to estimate survival. We used a 2-sample *t*-test to determine if differences in survival occurred between male and female cubs, despite timing of birth. We ran Program MARK analyses with and without sex as a covariate to determine if sex was related to survival. We analyzed cub survival in 2 ways. First, we placed cubs in categories; within or outside of the birth pulse to replicate the analysis conducted by Laundré and Hernández [4]. Second, we delineated the birth pulse according to our description of the predator-avoidance strategy switch, which provided a more detailed examination.

To investigate the role of mother's age on birth timing, we calculated the monthly frequency distribution of litters born by mother's age (i.e., 3–4 yrs, 4–6 yrs, >6 yrs). We also calculated the monthly frequency distribution of litters born by litter sequence (i.e., 1^st^ litter, >1^st^ litter). We visually compared birth month frequencies by mother's age and litter sequence.
